# Venetoclax enhances DNA damage induced by XPO1 inhibitors: A novel mechanism underlying the synergistic antileukaemic effect in acute myeloid leukaemia

**DOI:** 10.1111/jcmm.17274

**Published:** 2022-03-31

**Authors:** Hanxi Yu, Shuangshuang Wu, Shuang Liu, Xinyu Li, Yuqing Gai, Hai Lin, Yue Wang, Holly Edwards, Yubin Ge, Guan Wang

**Affiliations:** ^1^ 12510 National Engineering Laboratory for AIDS Vaccine Key Laboratory for Molecular Enzymology and Engineering the Ministry of Education School of Life Sciences Jilin University Changchun China; ^2^ Department of Hematology and Oncology the First Hospital of Jilin University Changchun China; ^3^ Department of Pediatric Hematology and Oncology the First Hospital of Jilin University Changchun China; ^4^ 12267 Department of Oncology and Molecular Therapeutics Program Barbara Ann Karmanos Cancer Institute Wayne State University School of Medicine Detroit Michigan USA

**Keywords:** acute myeloid leukaemia, combination treatment, DNA damage, venetoclax, XPO1 inhibitor

## Abstract

Acute myeloid leukaemia (AML) is a highly heterogeneous haematologic malignancy with poor prognosis. We previously showed synergistic antileukaemic interaction between exportin 1 (XPO1) inhibitor KPT‐330 (Selinexor) and Bcl‐2 inhibitor venetoclax (ABT‐199) in preclinical models of AML, which was partially meditated by Mcl‐1, although the full mechanism of action remains unknown. In this study, using real‐time RT‐PCR and Western blot analysis, we show that inhibition of XPO1 via KPT‐330 or KPT‐8602 (Eltanexor) decreases the mRNA and protein levels of c‐Myc, CHK1, WEE1, RAD51 and RRM2. KPT‐330 and KPT‐8602 induce DNA damage, as determined by alkaline comet assay. In addition, we demonstrate that venetoclax enhances KPT‐330‐ and KPT‐8602‐induced DNA damage, likely through inhibition of DNA damage repair. This study provides new insight into the molecular mechanism underlying the synergistic antileukaemic activity between venetoclax and XPO1 inhibitors against AML. Our data support the clinical evaluation of this promising combination therapy for the treatment of AML.

## 1 INTRODUCTION

Acute myeloid leukaemia (AML) is a hematopoietic disorder characterized by numerous cytogenetic and molecular aberrations, representing about 70% of adult acute leukaemia. Since the 1970s, the standard of care therapeutic for AML has been cytarabine and anthracycline based chemotherapy. The five‐year overall survival rate of adult patients with AML is only 26%, and less than 10% in patients over 60 years old.^1^, ^2^ Therefore, there is an urgent need for new therapies to improve the survival rate and quality of life for patients with AML. Venetoclax (ABT‐199) is a highly potent, orally bioavailable Bcl‐2 selective inhibitor. It was approved by the U.S. Food and Drug Administration (FDA) in November 2018 for the treatment of newly diagnosed AML patients at age 75 or elder, or who have comorbidities which preclude the use of intensive induction chemotherapy, in combination with azacitidine, decitabine or low dose cytarabine.^3^ To further improve the antileukaemic efficacy and applicability of venetoclax, combining it with other targeting inhibitors is a feasible strategy.

Exportin 1 (XPO1), also known as chromosome region maintenance 1 (CRM1), is a protein transporter responsible for over 220 cargo proteins including tumour suppressor proteins (TSPs) and RNAs to across the nuclear envelope to the cytoplasm.^4^, ^5^ Dysregulation of XPO1‐mediated nuclear export is evident in several haematologic malignancies and solid tumours, leading to enhanced transport of TSPs out of the nucleus.^6^, ^7^ KPT‐330 (Selinexor) is one of the first‐generation orally bioavailable selective inhibitors of XPO1,^8^, ^9^ and its combination with dexamethasone was granted accelerated FDA approval for adult patients with relapsed or refractory multiple myeloma in July 2019.^10^ KPT‐330 is also undergoing clinical development in a wide range of haematologic malignancies and solid tumours.^11^, ^12^ The second‐generation XPO1 inhibitor KPT‐8602 (Eltanexor) has similar mechanism of action and potency as KPT‐330,^13^ but has greater tolerability than KPT‐330 due to its lower central nervous system penetration^14^ and exhibits promising antileukaemic activity against AML.^15^

Our previous study demonstrated that KPT‐330 could enhance the antileukaemic activity of venetoclax against AML in a synergistic manner at least partially through downregulation of Mcl‐1. However, overexpression of Mcl‐1 only moderately rescued the AML cells from the combination treatment of KPT‐330 and venetoclax.^16^ Therefore, additional factors underlying the molecular mechanism remain to be elucidated.

c‐Myc is an important transcription factor, regulating about 15% of genes in the whole genome.^17^ c‐Myc also plays a critical role in the survival of AML cells.^18^, ^19^ Several studies have demonstrated that KPT‐330 can downregulate c‐Myc.^20^, ^21^, ^22^ In addition, KPT‐330 has been shown to induce DNA damage in cancer cells.^23^ CHK1, WEE1 and RAD51 are important components of the DNA damage response (DDR) network.^24^ In addition, ribonucleotide reductase (RR) catalyses the reduction in ribonucleotides into corresponding deoxyribonucleotides which are critical in DNA replication and DNA damage repair, thus plays a crucial role in maintaining genome stability.^25^ Previous studies have shown that c‐Myc has potential regulatory effects on CHK1, WEE1, RAD51 and RR.^21^, ^26^ Thus, it is conceivable that inhibition of XPO1 induces DNA damage in AML cells through downregulation of c‐Myc, CHK1, WEE1, RAD51 and RR.

In addition, we have previously reported that venetoclax can enhance the antileukaemic activity of DNA damaging drugs via enhancing DNA damage induced by these agents, leading to synergistic antileukaemic activity against AML.^27^ Based on this, we hypothesized that venetoclax would enhance XPO1 inhibitor‐induced DNA damage resulting in synergistic antileukaemic activity against AML cells.

## 2 MATERIALS AND METHODS

### 2.1 Drugs

KPT‐330, KPT‐8602, Z‐VAD‐FMK, 10058‐F4, MG‐132 and venetoclax were purchased from AbMole Bioscience (Shanghai branch, Shanghai, China).

### 2.2 Cell culture

MV4‐11 and THP‐1 AML cell lines were purchased from the American Type Culture Collection (Manassas, VA, USA). OCI‐AML3 was purchased from the German Collection of Microorganisms and Cell Cultures (DSMZ, Braunschweig, Germany). The cell lines were cultured as previously described^28^ and authenticated by Genetic Testing Biotechnology Corporation (Suzhou, China) in 2021. Mycoplasma testing was performed monthly, by PCR.^29^

### 2.3 Clinical samples

Diagnostic AML patient samples were obtained from the First Hospital of Jilin University following written informed consent was obtained based on the Declaration of Helsinki. This study was approved by and carried out in accordance with the guidelines as set forth by the Human Ethics Committee of the First Hospital of Jilin University. Primary AML patient cells were isolated and screened for the presence of both gene mutations and fusion genes as described previously.^30^ Samples were chosen based on availability of adequate sample at the time the assay was performed. Patient characteristics are shown in Table S1.

### 2.4 Western blot analysis

AML cells were lysed by sonication in 10 mM Tris‐Cl, pH 7.0, containing 1% SDS, protease inhibitors and phosphatase inhibitors (Roche Diagnostics, Indianapolis, IN, USA). Whole‐cell lysates were subjected to SDS‐polyacrylamide gel electrophoresis, electrophoretically transferred onto polyvinylidene difluoride (PVDF) membranes (Thermo Fisher Scientific, Rockford, IL, USA) and immunoblotted with antibodies. Anti‐γH2AX, anti‐H4 (Millipore, Billerica, MA, USA), anti‐WEE1, anti‐RAD51, anti‐p‐CDC25C(S216), anti‐cleaved (cf) caspase 3, anti‐c‐Myc, anti‐Bcl‐2, anti‐β‐actin (Proteintech Group, Chicago, IL, USA), anti‐CHK1, anti‐CDK2, anti‐MEK (Cell Signaling Technology, Danvers, MA, USA), anti‐p‐CDK1(Y15), anti‐CDK1, anti‐p‐CDK2(Y15), anti‐RRM1 and anti‐RRM2 (Abcam, Cambridge, MA, USA) antibodies were used for Western blot analysis. Immunoreactive proteins were visualized using the Odyssey Infrared Imaging System (Li‐Cor, Lincoln, NE, USA), as described by the manufacturer. The fold changes in protein densitometry measurements were normalized to β‐actin and then compared with the vehicle control.

### 2.5 Annexin V‐FITC/PI staining and flow cytometry analysis

AML cells were treated with vehicle, KPT‐330, KPT‐8602, venetoclax and venetoclax plus KPT‐330 or KPT‐8602, and then underwent flow cytometry analysis utilizing the Annexin V‐fluorescein isothiocyanate (FITC)/propidium iodide (PI) apoptosis kit (Beckman Coulter; Brea, CA, USA) to determine the extent of drug‐induced apoptosis, as previously described.^28^ Annexin V+/PI‐ and Annexin V+/PI+cells represent early apoptotic and late apoptotic (dead) cells respectively. Results are shown as mean percentage of Annexin V‐positive cells ± the standard error of the mean (SEM) of replicates from two independent experiments. Cells treated with an apoptosis inducer purchased from Beyotime Biotechnology (Shanghai, China) were used as the positive control.

### 2.6 Real‐time RT‐PCR

Total RNA was extracted using TRIzol (Life Technologies, Grand Island, NY, USA). cDNAs were prepared from 1 µg total RNA using random hexamer primers and a RT‐PCR kit (Thermo Fisher Scientific), and then purified using the QIAquick PCR Purification Kit (Qiagen, Germantown, MD, USA), as described previously.^28^ CHK1 (Hs00967506_m1), RAD51 (Hs00153418_m1) and WEE1 (Hs01119384_g1) transcripts were quantitated using TaqMan probes (Thermo Fisher Scientific). RRM2 transcripts were quantified using forward primer (5'‐TGGTCGACAAGGAGAACACG‐3’), reverse primer (5'‐TTAGTTTTCGGCTCCGTGGG‐3’) and SYBR green (Thermo Fisher Scientific). c‐Myc transcripts were quantified using forward primer (5'‐GTGGTCTTCCCCTACCCTCT‐3’), reverse primer (5'‐CGAGGAGAGCAGAGAATCCG‐3’) and SYBR green. Analyses were performed by a LightCycler 480 real‐time PCR machine (Roche Diagnostics), based on the manufacturer's instructions. Ct values were normalized to GAPDH transcripts measured by either TaqMan probe (Hs02786624_g1) or forward primer (5'‐AGCCACATCGCTCAGACA‐3’), reverse primer (5'‐GCCCAATACGACCAAATCC‐3’) and SYBR green. Fold changes were calculated using the comparative Ct method.^31^ Data are presented as mean of replicates ±SEM from two independent experiments.

### 2.7 Alkaline comet assay

AML cells were subjected to alkaline comet assay, as previously described.^28^ Slides were stained using SYBR Gold (Life Technologies) and then imaged by an Olympus BX‐40 microscope equipped with a DP72 microscope camera and Olympus CellSens Dimension software (Olympus America Inc., Center Valley, PA, USA). Approximately 50 comets were scored per gel, using CometScore (TriTek Corp, Sumerduck, VA, USA). Median per cent DNA in the tail from three gels was calculated and graphed as mean ± the standard error.

### 2.8 Nucleus and cytoplasm fractionation

Nucleus and cytoplasm fractionation was carried out as described by Buisson and colleagues.^32^ 3 × 10^6^ cells were washed with PBS and resuspended in solution A (10 mM HEPES, pH 7.9, 10 mM KCl, 1.5 mM MgCl_2_, 0.34 M sucrose, 10% glycerol, 1 mM DTT, 10 mM NaF, 1 mM Na_2_VO_3_ and protease inhibitors purchased from Roche Diagnostics). Triton X‐100 was added (final concentration of 0.1%), and then, the cells were incubated on ice for 4~5 min. Nuclei were separated from cytoplasmic proteins by centrifugation at 1400× g for 4 min and then washed with solution A three times. Nuclei were resuspended and sonicated, and then subjected to Western blot analysis. Experiments were repeated three times independently. Representative blots are shown. The fold changes in protein densitometry measurements were compared with histone H4 in nuclear fraction or MEK in cytoplasmic fraction and then normalized to the vehicle control. Data are presented as mean ± SEM.

### 2.9 Statistical analysis

Differences in cellular apoptosis (the sum of Annexin V+ cells), the fold change in transcript and the per cent DNA present in the tail were compared by an unpaired two‐sample t‐test. Statistical analyses were performed with GraphPad Prism 5.0 (GraphPad Software, LaJolla, CA, USA). Error bars represent ±standard error of the mean. The significance level was set at *p* < 0.05.

## 3 RESULTS

### 3.1 The effect of XPO1 inhibition on c‐Myc and DDR proteins in AML cell lines

**FIGURE 1:**
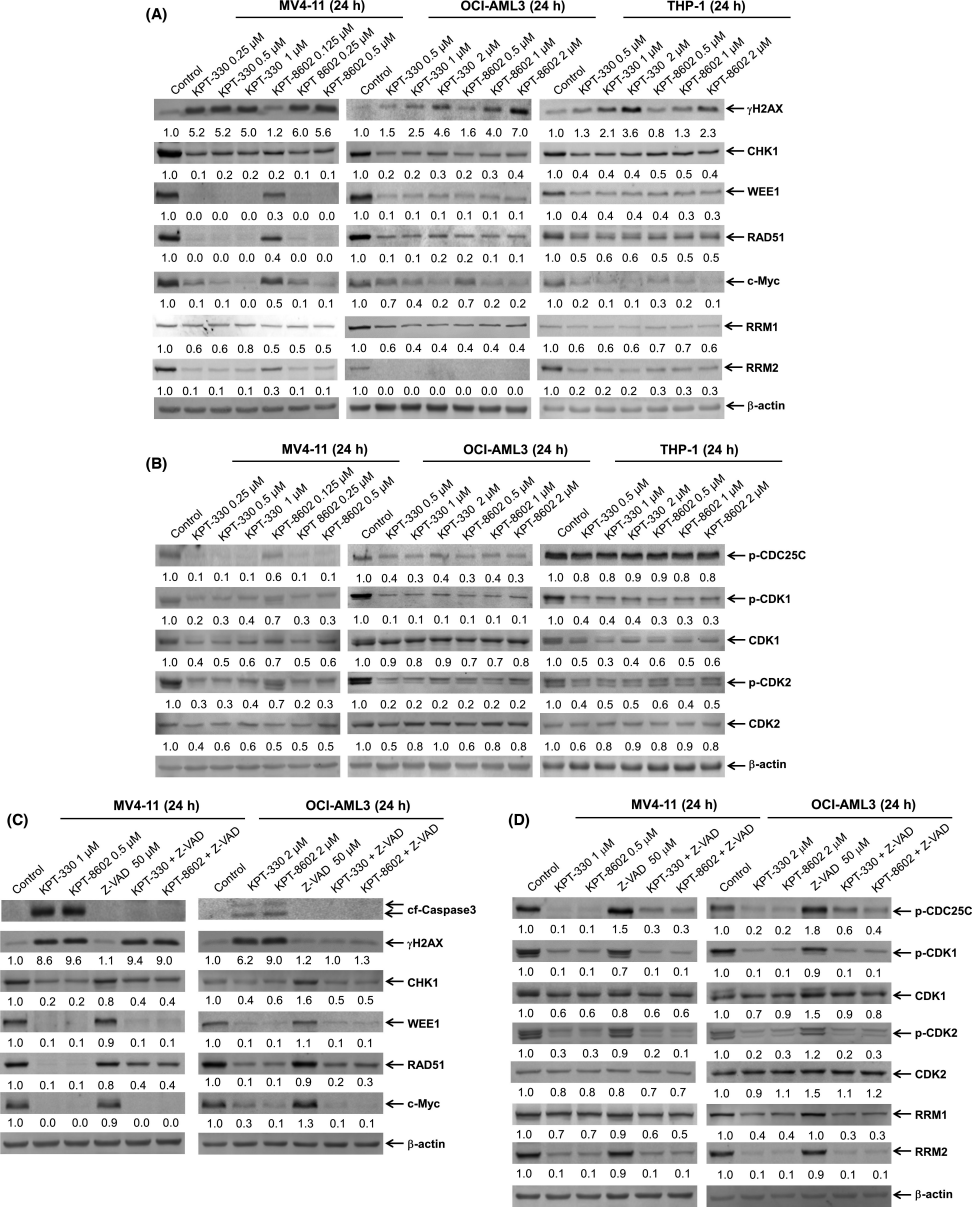
Inhibition of XPO1 downregulates DDR pathway proteins in AML cells. (A and B) MV4‐11, OCI‐AML3 and THP‐1 cells were treated with KPT‐330 or KPT‐8602 for 24 h. Whole‐cell lysates were subjected to Western blotting and probed with the indicated antibodies. The fold changes for the densitometry measurements, normalized to β‐actin and then compared with vehicle control, are graphed below the corresponding blot. (C and D) MV4‐11 and OCI‐AML3 cells were treated with KPT‐330, KPT‐8602 and Z‐VAD‐FMK (Z‐VAD) alone or in combination, for 24 h. Whole‐cell lysates were subjected to Western blot analysis and probed with the indicated antibodies. The fold changes for the densitometry measurements, normalized to β‐actin and then compared with vehicle control, are graphed below the corresponding blot

KPT‐330 has been reported to downregulate DNA repair proteins leading to DNA damage in tumour cells, including AML cells.^23^ Based on reports that KPT‐330 can downregulate c‐Myc^20^, ^21^, ^22^ and c‐Myc has potential regulatory effects on CHK1, WEE1, RAD51 and RR,^21^, ^26^ we suspected that these may be downregulated by XPO1 inhibition. RR is composed of a regulatory subunit RRM1 and a catalytic subunit RRM2.^33^ Thus, we investigated the effect of XPO1 inhibition on γH2AX (an established biomarker for DNA double‐strand breaks^34^), c‐Myc, CHK1, WEE1, RAD51, RRM1 and RRM2 protein levels. We treated MV4‐11, OCI‐AML3 and THP‐1 cells with various concentrations of KPT‐330 or KPT‐8602 for 24 h. Whole‐cell lysates were subjected to Western blot analysis. KPT‐330 and KPT‐8602 treatment increased γH2AX levels, indicating increased DNA double‐strand breaks (Figure 1A). In addition, KPT‐330 and KPT‐8602 treatment caused downregulation of c‐Myc, CHK1, WEE1, RAD51, RRM1 and RRM2 (Figure 1A). Consistent with the decrease in CHK1 and WEE1, decreased phosphorylation of CDC25C (Ser216), CDK1 (Tyr15) and CDK2 (Tyr15) in MV4‐11 and OCI‐AML3 cells and downregulation of p‐CDK1 and p‐CDK2 in THP‐1 cells were detected post KPT‐330 and KPT‐8602 treatment (Figure 1B). Previously, we demonstrated that KPT‐330 and KPT‐8602 induce apoptosis under these treatment conditions^16^; thus, the downregulation of these proteins might be due to caspase cleavage. To rule out caspase‐dependent cleavage, we treated MV4‐11 and OCI‐AML3 cells with KPT‐330 or KPT‐8602 alone or in combination with the pan‐caspase inhibitor Z‐VAD‐FMK for 24 h. Cleavage of caspase 3 induced by KPT‐330 and KPT‐8602 treatment was completely abolished by Z‐VAD‐FMK. Although KPT‐330‐ and KPT‐8602‐induced‐γH2AX was unaffected by Z‐VAD‐FMK in MV4‐11 cells, Z‐VAD‐FMK eliminated induction of γH2AX by KPT‐330 and KPT‐8602 in OCI‐AML3 cells, suggesting that the increase in γH2AX was indicative of cell death in OCI‐AML3 cells. However, Z‐VAD‐FMK had little effect on KPT‐330‐ and KPT‐8602‐induced downregulation of c‐Myc, CHK1, WEE1, RRM1, RRM2, p‐CDC25C, p‐CDK1 and p‐CDK2 in MV4‐11 and OCI‐AML3 cells (Figure 1C,D). RAD51 downregulation was partially rescued by the addition of Z‐VAD‐FMK in both MV4‐11 and OCI‐AML3 cells. Taken together, these results demonstrate that inhibition of XPO1 results in decrease in c‐Myc and some DDR proteins, independent of caspase activation.

**FIGURE 2:**
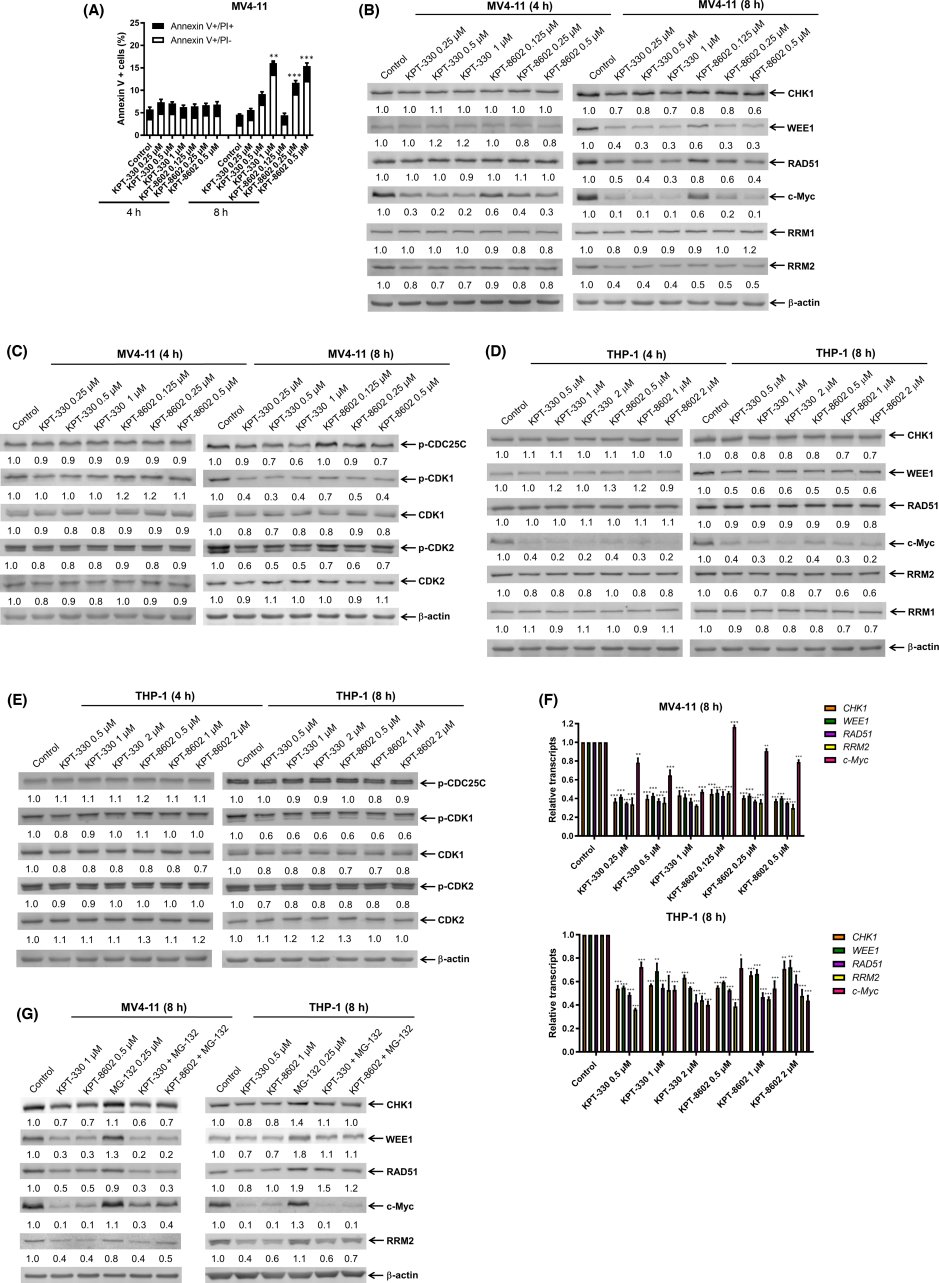
Effect of short‐term inhibition of XPO1 on the protein and mRNA levels of c‐Myc and DDR pathway components in AML cells. (A) MV4‐11 cells were treated with KPT‐330 or KPT‐8602, for 4 or 8 h, and then subjected to Annexin V‐FITC/PI staining and flow cytometry analysis. Mean per cent Annexin V+ cells ± SEM are shown. ** indicates *p* < 0.01 and *** indicates *p* < 0.001 compared with vehicle control. (B–E) MV4‐11 and THP‐1 cells were treated with KPT‐330 or KPT‐8602, for 4 or 8 h. Whole‐cell lysates were subjected to Western blot analysis and probed with the indicated antibodies. The fold changes for the densitometry measurements, normalized to β‐actin and then compared with vehicle control, are graphed below the corresponding blot. (F) MV4‐11 and THP‐1 cells were treated with KPT‐330 or KPT‐8602, for 8 h. Total RNA was extracted and *c*‐*Myc*, *CHK1*, *WEE1*, *RAD51*, *RRM2* and *GAPDH* transcripts were determined by real‐time RT‐PCR. The relative changes in transcripts, normalized to GAPDH, in comparison with control samples were quantified. * indicates *p* < 0.05, ** indicates *p* < 0.01, and *** indicates *p* < 0.001 compared with vehicle control. (G) MV4‐11 and THP‐1 cells were treated with KPT‐330, KPT‐8602 and MG‐132, alone or in combination, for 8 h, and then subjected to Western blot analysis and probed with the indicated antibodies. The fold changes for the densitometry measurements, normalized to β‐actin and then compared with vehicle control, are graphed below the corresponding blot

To further determine whether the downregulation of c‐Myc and these DDR proteins occurs prior to induction of apoptosis, MV4‐11 cells were treated with KPT‐330 and KPT‐8602 for 4 and 8 hours. Increased Annexin V positivity was detected after 8 h of treatment, although the magnitude for the lower concentrations was minimal (Figure 2A). Downregulation of c‐Myc was detected at 4 h, while downregulation of CHK1, WEE1, RAD51 and RRM2 was detected at 8 h (Figure 2B). Consistent with the decrease in CHK1 and WEE1, decreased phosphorylation of CDC25C (Ser216), CDK1 (Tyr15) and CDK2 (Tyr15) in MV4‐11 cells was detected post 8‐h KPT‐330 and KPT‐8602 treatment (Figure 2C). RRM1 protein levels remained relatively unchanged. In THP‐1 cells, 8‐h KPT‐330 and KPT‐8602 treatment had no obvious effect on apoptosis (Figure S1). Consistent with MV4‐11 cells, c‐Myc downregulation was detected at 4 h, and downregulation of CHK1, WEE1, RRM2, p‐CDK1 and p‐CDK2 was detected at 8 h in THP‐1 cells (Figure 2D and E). RRM1 downregulation was detected at 8 h in THP‐1 cells. Phosphorylation of CDC25C remained unchanged after treatment with either XPO1 inhibitor. These results show that inhibition of XPO1 downregulates c‐Myc prior to induction of apoptosis, and that downregulation of CHK1, WEE1 and RRM2 occurs at least prior to mass apoptosis. Due to the lack of obvious decrease post KPT‐330 and KPT‐8602 treatment, RRM1 was excluded for the rest of this study.

### 3.2 Inhibition of XPO1 decreases the mRNA levels of c‐Myc, CHK1, WEE1, RAD51 and RRM2

Since c‐Myc downregulation occurs prior to downregulation of CHK1, WEE1, RAD51 and RRM2 and it transcriptionally regulates CHK1, WEE1, RAD51 and RRM2,^21^, ^26^ we measured their mRNA levels following 8‐h treatment with KPT‐330 or KPT‐8602. We found that *c‐Myc*, *CHK1*, *WEE1*, *RAD51* and *RRM2* mRNA levels were significantly decreased by KPT‐330 and KPT‐8602 treatment in both cell lines, although the lowest concentration of KPT‐8602 upregulated *c‐Myc* mRNA for unknown reasons in MV4‐11 cells (Figure 2F). These results indicate that decreased transcripts play a role in the downregulation of the corresponding proteins. To determine whether the proteasome pathway plays a role in KPT‐330 and KPT‐8602 downregulation of c‐Myc, CHK1, WEE1, RAD51 and RRM2, MV4‐11 and THP‐1 cells were treated with KPT‐330, KPT‐8602 and proteasome inhibitor MG‐132, alone or in combination for 8 h. Western blot analysis revealed that MG‐132 had little‐to‐no effect on KPT‐330 and KPT‐8602 downregulation of CHK1, WEE1, RAD51 and RRM2 in MV4‐11 cells and c‐Myc in THP‐1 cells (Figure 2G). Otherwise, c‐Myc protein level in MV4‐11 cells and CHK1, WEE1, RAD51 and RRM2 protein levels in THP‐1 cells were slightly increased by the addition of MG‐132 to the XPO1 inhibitors. These results suggest that KPT‐330 and KPT‐8602 downregulate *c‐Myc*, *CHK1*, *WEE1*, *RAD51* and *RRM2* transcripts, although proteasome degradation may also play a role.

### 3.3 Inhibition of XPO1 induces DNA damage

**FIGURE 3:**
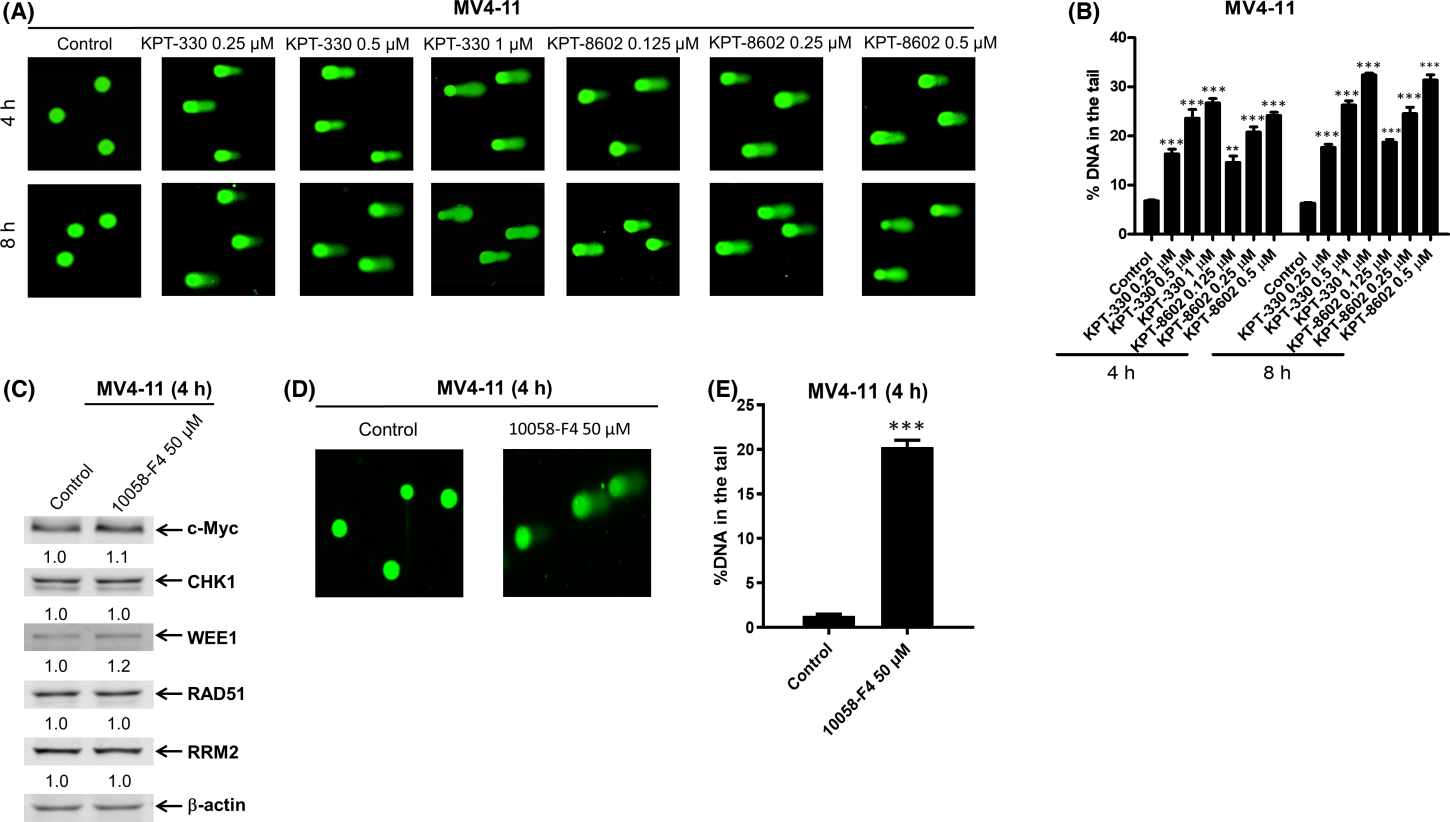
Inhibition of XPO1 or c‐Myc could induce DNA damage in AML cells. (A) MV4‐11 cells were treated with KPT‐330 or KPT‐8602, for 4 or 8 h, and then subjected to alkaline comet assay. Representative images from alkaline comet assay are shown in panel A. Alkaline comet assay results are graphed as median per cent DNA in the tail from three replicate gels ± SEM (panel B). (C) MV4‐11 cells were treated with 10058‐F4 for 4 h, and then subjected to Western blot analysis or alkaline comet assay. Whole‐cell lysates were subjected to Western blot analysis and probed with the indicated antibodies. The fold changes for the densitometry measurements, normalized to β‐actin and then compared with vehicle control, are graphed below the corresponding blot (panel C). Representative images from the alkaline comet assay are shown in panel D. Alkaline comet assay results are graphed as median per cent DNA in the tail from three replicate gels ± SEM (panel E). *** indicates *p* < 0.001 compared with vehicle control

Given the reported role in DNA repair,^24^, ^35^ downregulation of c‐Myc and DDR proteins likely induces DNA damage. To test this possibility, MV4‐11 and THP‐1 cells were treated with KPT‐330 and KPT‐8602 for 4 and 8 h, and then subjected to alkaline comet assay. KPT‐330 and KPT‐8602 treatment induced DNA damage, as measured by the per cent DNA in the comet tails, in a concentration‐dependent manner (Figure 3Aa,B and Figure S1B and C). Importantly, increased DNA damage was detected prior to cell apoptosis (Figure 2A and Figure S1A). These results demonstrate that inhibition of XPO1 induces DNA damage in AML cells.

### 3.4 Inhibition of c‐Myc induces DNA damage without downregulating CHK1, WEE1, RAD51 and RRM2

Since inhibition of XPO1 for 4 h downregulated c‐Myc and induced DNA damage, but had little effect on CHK1, WEE1, RAD51 and RRM2 (Figure 2B,D, Figure 3A,B and Figure S1B and C), we suspected that downregulation of c‐Myc induced DNA damage. To test this, we investigated the effect of c‐Myc inhibition on DNA damage. MV4‐11 and THP‐1 cells were treated with c‐Myc inhibitor 10058‐F4^36^ for 4 h and then subjected to Western blotting analysis, real‐time RT‐PCR and alkaline comet assay respectively. We found that pharmacologically inhibiting c‐Myc induced significant DNA damage but had little effect on the protein levels of CHK1, WEE1, RAD51 and RRM2 (Figure 3C–E and Figure S1D–F). These results suggest that decrease in c‐Myc plays an important role in the DNA damage induced by XPO1 inhibition.

### 3.5 The effect of XPO1 inhibition on the DDR proteins and DNA damage in AML patient samples

**FIGURE 4:**
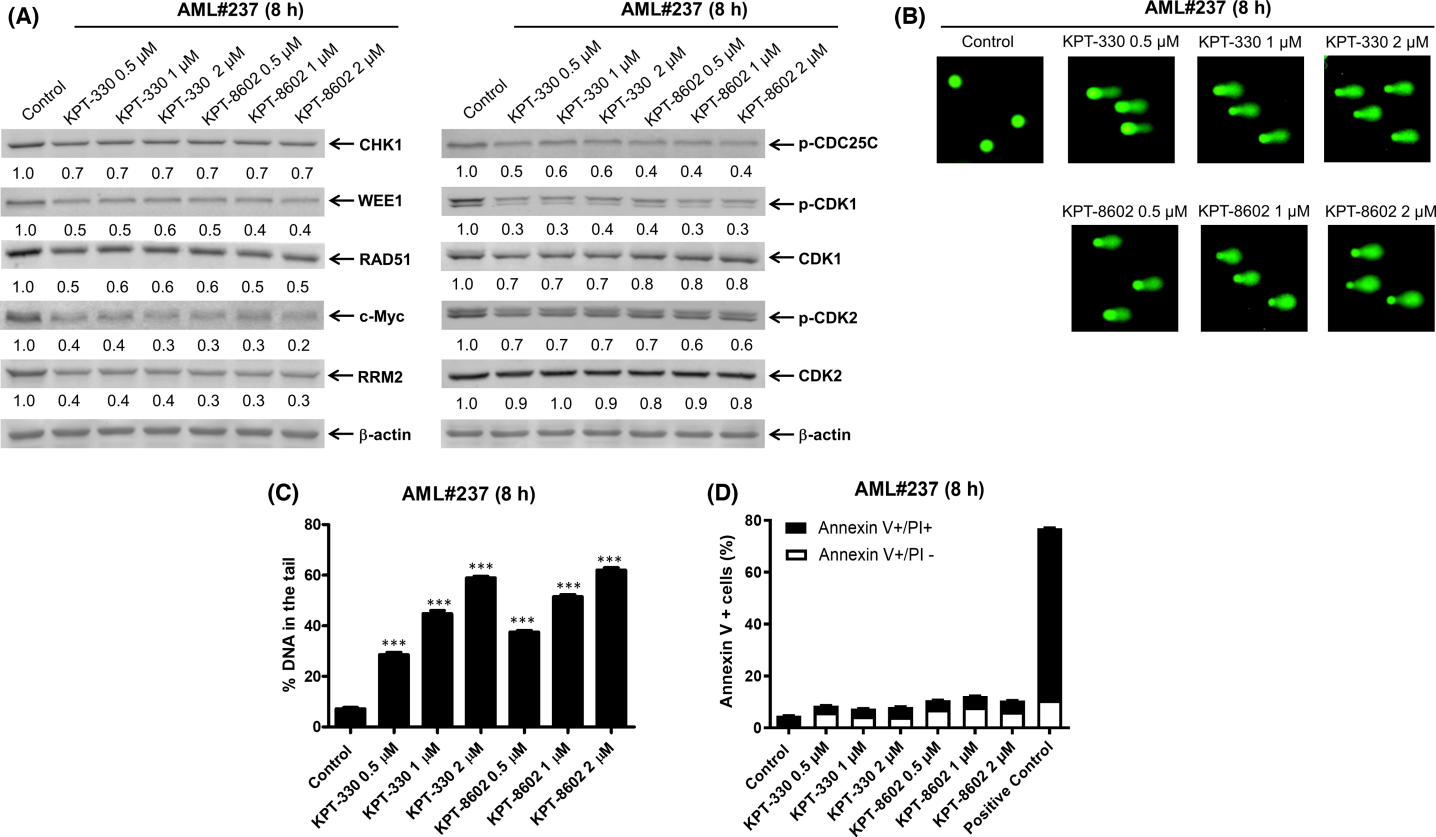
Effect of XPO1 inhibition on c‐Myc, DDR pathway proteins and DNA damage in AML patient samples. AML patient sample cells were treated with KPT‐330 or KPT‐8602 for 8 h and then subjected to Western blot analysis, alkaline comet assay or flow cytometry analysis. (A) The levels of the indicated proteins were analysed by Western blot analysis. The fold changes for the densitometry measurements, normalized to β‐actin and then compared with vehicle control, are graphed below the corresponding blot. (B) Representative images from alkaline comet assay are shown. (C) Alkaline comet assay results are graphed as median per cent DNA in the tail from three replicate gels ± SEM. *** indicates *p* < 0.001 compared with vehicle control. (D) Annexin V‐FITC/PI staining was assessed by flow cytometry analysis. Mean per cent Annexin V+ cells ± SEM are shown. Cells treated with an apoptosis inducer purchased from the Beyotime Biotechnology (Shanghai, China) were used as the positive control

To determine whether the effects of KPT‐330 and KPT‐8602 on DNA damage, c‐Myc and DDR proteins also occur in primary AML patient samples, we treated primary AML patient samples AML#237 and AML#213 with KPT‐330 and KPT‐8602 for 8 h. Consistent with the AML cell lines, downregulation of CHK1, WEE1, RAD51, c‐Myc, RRM2, p‐CDC25C, p‐CDK1 and p‐CDK2 was detected in both patient samples (Figure 4A and Figure S2A). KPT‐330 and KPT‐8602 treatment for 8 h induced DNA damage (Figure 4B and C and S2B and C). Annexin V/PI staining and flow cytometry analysis revealed that 8‐h treatment with KPT‐330 or KPT‐8602 did not significantly induce apoptosis in AML#237. Due to the limited cell number, apoptosis analysis was not performed in AML#213. These data confirm the results obtained in AML cell lines and demonstrate that inhibition of XPO1 can induce downregulation of c‐Myc, the DDR proteins and DNA damage in primary AML patient samples.

### 3.6 Venetoclax enhances DNA damage induced by inhibition of XPO1

**FIGURE 5:**
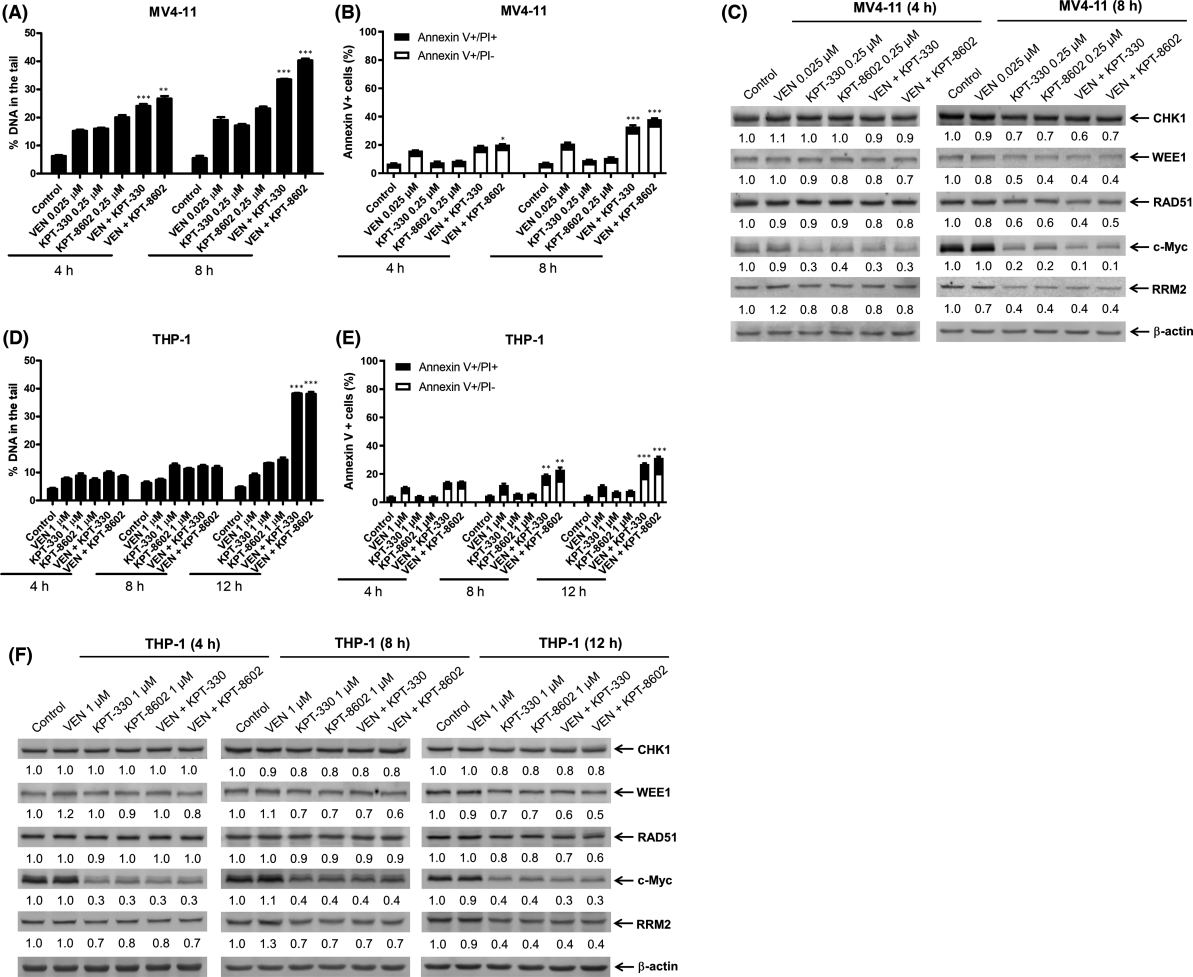
Venetoclax enhances DNA damage induced by XPO1 inhibition in AML cells. MV4‐11 and THP‐1 cells were treated with KPT‐330, KPT‐8602 and venetoclax (VEN), alone or in combination for 4, 8 or 12 h, and then subjected to alkaline comet assay, flow cytometry analysis or Western blot analysis. (A and D) Alkaline comet assay results are graphed as median per cent DNA in the tail from three replicate gels ± SEM. ** indicates *p* < 0.01 and *** indicates *p* < 0.001 compared with single agent treatment. (B and E) Annexin V‐FITC/PI staining was assessed by flow cytometry analysis. Mean per cent Annexin V+ cells ± SEM are shown. * indicates *p* < 0.05, ** indicates *p* < 0.01, and *** indicates *p* < 0.001 compared with single agent treatment. (C and F) Western blots of whole cell lysates were probed with the indicated antibodies. The fold changes for the densitometry measurements, normalized to β‐actin and then compared with vehicle control, are graphed below the corresponding blot

We previously demonstrated that venetoclax impairs the repair of DNA damage induced by DNA damaging agents.^27^, ^37^ Thus, we hypothesized that venetoclax would enhance the DNA damage induced by XPO1 inhibition. To begin to test this hypothesis, we treated MV4‐11 and THP‐1 cells with KPT‐330, KPT‐8602, venetoclax, KPT‐330 + venetoclax or KPT‐8602 + venetoclax. Combination treatment induced significantly more DNA damage compared with single agent treatment and was accompanied by a low level of apoptosis (Figure 5A,B,D,E). Treatment with venetoclax had limited effect on the protein level of c‐Myc, CHK1, WEE1, RAD51 and RRM2. KPT‐330 or KPT‐8602 combined with venetoclax decreased c‐Myc, CHK1, WEE1, RAD51 and RRM2, similar to that of KPT‐330 and KPT‐8602 treatment alone (Figure 5C,F). The results show that venetoclax enhances DNA damage induced by XPO1 inhibition without causing further downregulation of c‐Myc, CHK1, WEE1, RAD51 and RRM2 induced by XPO1 inhibition.

**FIGURE 6:**
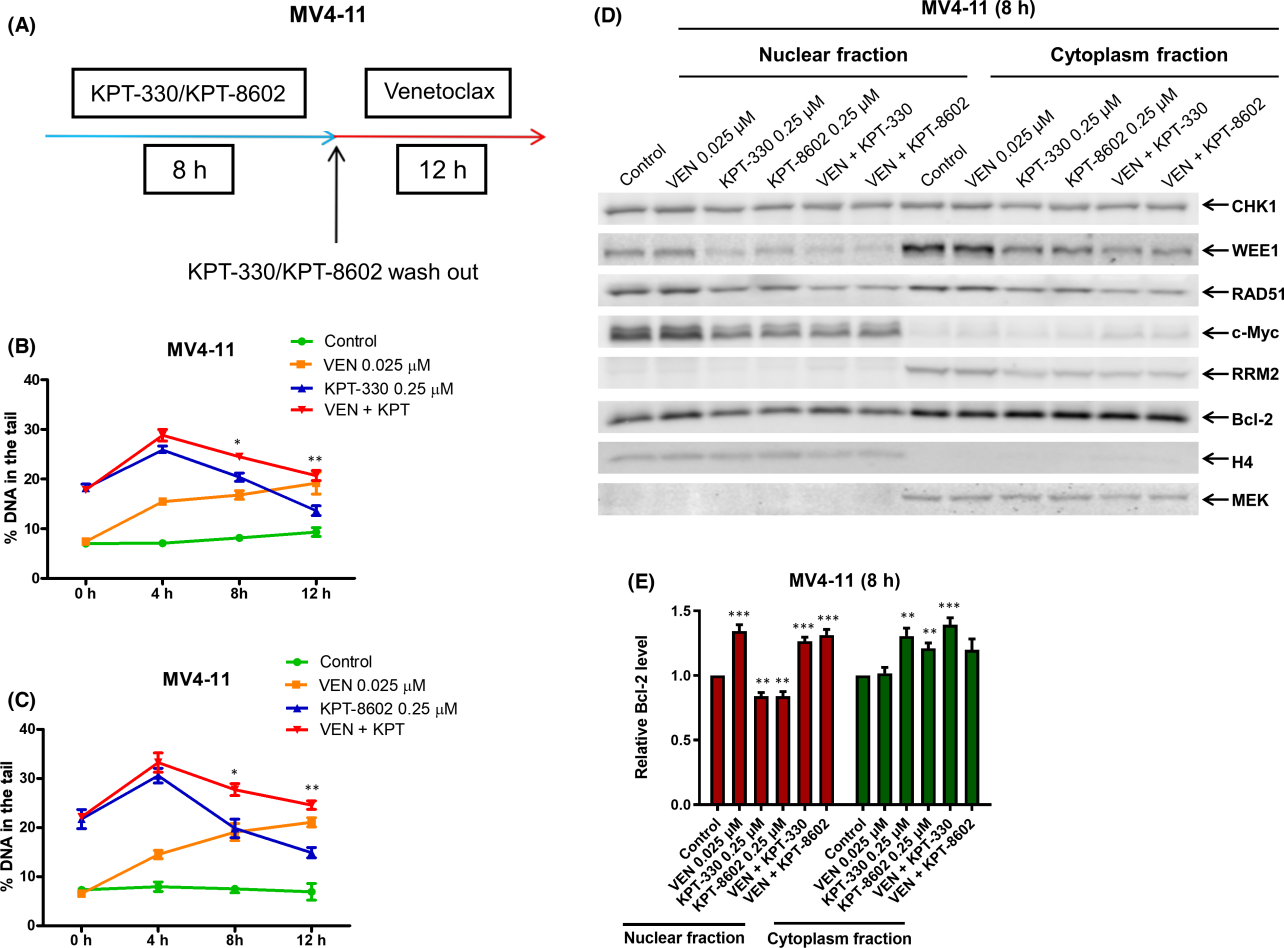
Venetoclax interferes with the repair of XPO1 inhibition‐induced DNA damage. (A‐C) MV4‐11 cells were treated with vehicle control, KPT‐330 or KPT‐8602 for 8 h. The cells were washed with PBS three times and then split, half receiving fresh media and the other half receiving fresh media plus venetoclax. Cells were collected at 0, 4, 8 and 12 h after addition of venetoclax. Alkaline comet assay results are shown as median per cent DNA in tail from three replicate gels ± SEM. * indicates *p* < 0.05 and ** indicates *p* < 0.01 compared with single agent treatment. (D and E) MV4‐11 cells were treated with KPT‐330, KPT‐8602 and venetoclax, alone or in combination for 8 h. Nuclear and cytoplasmic fractions were extracted and subjected to Western blotting. The fold changes for the Bcl‐2 densitometry measurements, normalized to H4 or MEK and then compared with vehicle control, are graphed as median per cent ± SEM in panel E. ** indicates *p* < 0.01 and *** indicates *p* < 0.001 compared with vehicle control

Next, we investigated the effect of venetoclax on the repair of DNA damage induced by KPT‐330 and KPT‐8602. Following exposure to KPT‐330 or KPT‐8602 for 8 h, MV4‐11 cells were washed and cultured in fresh media (KPT‐330 and KPT‐8602 free) with or without venetoclax for up to 12 h, and then subjected to alkaline comet assay (Figure 6A). The level of DNA damage decreased after removal of KPT‐330 and KPT‐8602, suggesting that DNA repair progressed after removal of KPT‐330 and KPT‐8602. Venetoclax treatment resulted in time‐dependent induction of DNA damage. Furthermore, 8 and 12 h after addition of venetoclax, significantly more DNA damage was detected in cells pretreated with KPT‐330 or KPT‐8602 in the presence of venetoclax compared with those without venetoclax (Figure 6B,C), indicating that venetoclax interfered with repair of DNA damage induced by XPO1 inhibition.

Regulating the nucleo‐cytoplasmic localization of proteins is the fundamental function of XPO1. In addition, it is worth noting that several studies have shown that Bcl‐2 can enter the nucleus and inhibit DNA repair.^38^, ^39^, ^40^ Thus, we examined the effects of XPO1 inhibition alone and in combination with venetoclax on the levels of c‐Myc, CHK1, WEE1, RAD51, RRM2 and Bcl‐2 in nucleus and cytoplasm. In MV4‐11 and THP‐1 cells, venetoclax alone and in combination with KPT‐330 or KPT‐8602 significantly increased the amount of Bcl‐2 in the nucleus, but had no obvious effect on the intracellular localization of c‐Myc, CHK1, WEE1, RAD51 and RRM2 (Figure 6D,E and Figure S3). These results suggest that venetoclax can impair DNA repair induced by XPO1 inhibition by enhancing Bcl‐2 nuclear localization in AML cells.

## 4 DISCUSSION

We previously reported that Mcl‐1 plays an important role in the antileukaemic activity of combined inhibition of XPO1 and Bcl‐2.^16^ In this study, we add further insight into the mechanism of action, the cooperative induction of DNA damage. Consistent with Ranganathan et al.,^21^ we found that KPT‐8602 downregulates c‐Myc, CHK1 and RAD51. They also found that KPT‐330 treatment decreases c‐Myc protein level and reduces c‐Myc binding to the promoters of CHK1 and RAD51 in AML cells.^21^ Furthermore, they reported that KPT‐330 induces DNA damage in cancer cells.^21^, ^23^ In contrast, we found that downregulation of c‐Myc and increased DNA strand breaks occurred prior to downregulation of CHK1 and RAD51. Additionally, we found that XPO1 inhibition also downregulated WEE1 and RRM2, which also occurred after detection of increased DNA damage. Interestingly, we found that c‐Myc inhibition significantly induced DNA damage (Figure 3D,E). These results suggest that c‐Myc plays an important role in DNA damage induced by XPO1 inhibition, while downregulation of CHK1, WEE1, RAD51 and RRM2 likely contributes at later time points. c‐Myc has been reported to be required for activation of checkpoints in response to DNA damage^35^ and evidence of transcription‐independent control of DNA replication.^41^ These mechanisms could contribute to the increase in DNA damage accompanied by downregulation of c‐Myc, although the exact mechanism remains unknown and beyond the scope of this study. Furthermore, WEE1 is a reported cargo protein of XPO1,^42^, ^43^ although we did not detect an increase in nuclear localized WEE1 following XPO1 inhibition. However, substantial downregulation of WEE1 protein following XPO1 inhibition may interfere with detecting a potential increase in the protein in the nucleus; thus, further studies are warranted. Our previous study showed that MV4‐11 cells are more sensitive to XPO1 inhibitors than THP‐1 cells.^16^ In this study, we found that treatment of THP‐1 cells with higher concentrations of XPO1 inhibitors induced much lesser DNA damage at a later time compared with that in MV4‐11 cells, indicating that the level and time of DNA damage induction by XPO1 inhibitors are potential determinants of their antileukaemic activity against AML.

In this study, we also found that XPO1 inhibition resulted in an increase in Bcl‐2 in the nuclear fraction (Figure 6D,E and Figure S3). Venetoclax is designed to target the hydrophobic cleft of Bcl‐2 composed of BH1, BH2 and BH3 domains.^44^ However, Bcl‐2 affects DNA repair via its BH4 domain.^39^, ^40^ Thus, our findings suggest that Bcl‐2 inhibition causes increased nuclear localization of the protein, resulting in inhibition of DNA repair. These results are consistent with our previous studies demonstrating that venetoclax impairs the repair of DNA damage induced by DNA damaging agents.^27^, ^37^

In conclusion, we demonstrate that inhibition of XPO1 causes downregulation of c‐Myc, CHK1, WEE1, RAD51 and RRM2 resulting in DNA damage and venetoclax can enhance DNA damage induced by XPO1 inhibition. Our findings further elucidate the antileukaemic mechanism of XPO1 inhibitors and venetoclax. Fischer et al. demonstrated that the combination of KPT‐8602 and venetoclax shows promising antileukaemic activity in both MV4‐11 cells and AML patient‐derived xenograft mouse models.^45^ Therefore, our current and past findings^16^ as well as those of other investigators^45^ provide preclinical support for further investigations into the clinical efficacy of this promising therapy for AML.

## CONFLICT OF INTEREST

The authors declare that they have no competing interests.

## AUTHOR CONTRIBUTIONS

**Hanxi Yu:** Formal analysis (equal); Investigation (lead); Validation (equal); Visualization (equal); Writing – original draft (equal); Writing – review & editing (equal). **Shuangshuang Wu:** Formal analysis (supporting); Investigation (supporting); Validation (supporting); Writing – review & editing (supporting). **Shuang Liu:** Formal analysis (supporting); Investigation (supporting); Validation (supporting); Writing – review & editing (supporting). **Xinyu Li:** Formal analysis (supporting); Investigation (supporting); Validation (supporting); Writing – review & editing (supporting). **Yuqing Gai:** Formal analysis (supporting); Investigation (supporting); Validation (supporting); Writing – review & editing (supporting). **Hai Lin:** Formal analysis (supporting); Resources (equal); Validation (supporting); Writing – review & editing (supporting). **Yue Wang:** Formal analysis (supporting); Resources (equal); Validation (supporting); Writing – review & editing (supporting). **Holly Edwards:** Formal analysis (supporting); Validation (supporting); Writing – original draft (equal); Writing – review & editing (equal). **Yubin Ge:** Formal analysis (supporting); Validation (supporting); Writing – review & editing (supporting). **Guan Wang:** Conceptualization (lead); Formal analysis (equal); Funding acquisition (lead); Methodology (lead); Project administration (lead); Supervision (lead); Validation (equal); Visualization (equal); Writing – original draft (equal); Writing – review & editing (equal).

## Supporting information

Fig S1

Fig S2

Fig S3

Table S1

Supplementary Material

## Data Availability

All data generated or analysed during this study are included in this published article and in the additional files.
