# Supplementary figures and images for: Venetoclax enhances DNA damage induced by XPO1 inhibitors: A novel mechanism underlying the synergistic antileukaemic effect in acute myeloid leukaemia
Source: J Cell Mol Med. 2022 Mar 31;26(9):2646–57. doi: 10.1111/jcmm.17274 (PMC9077288; doi:10.1111/jcmm.17274)

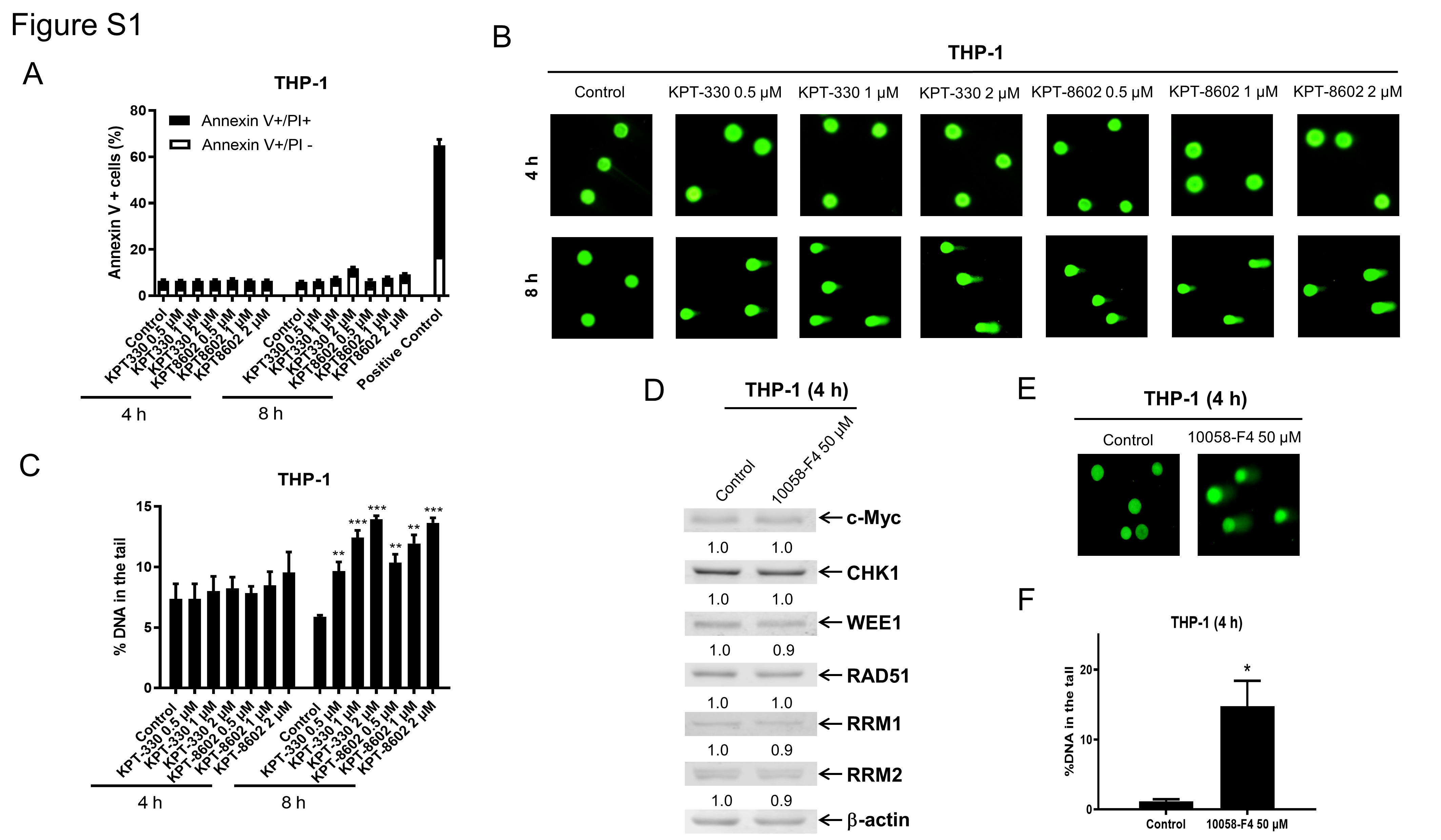

Supplement: Supplementary file 1 — Fig S1 [file JCMM-26-2646-s004.tif]

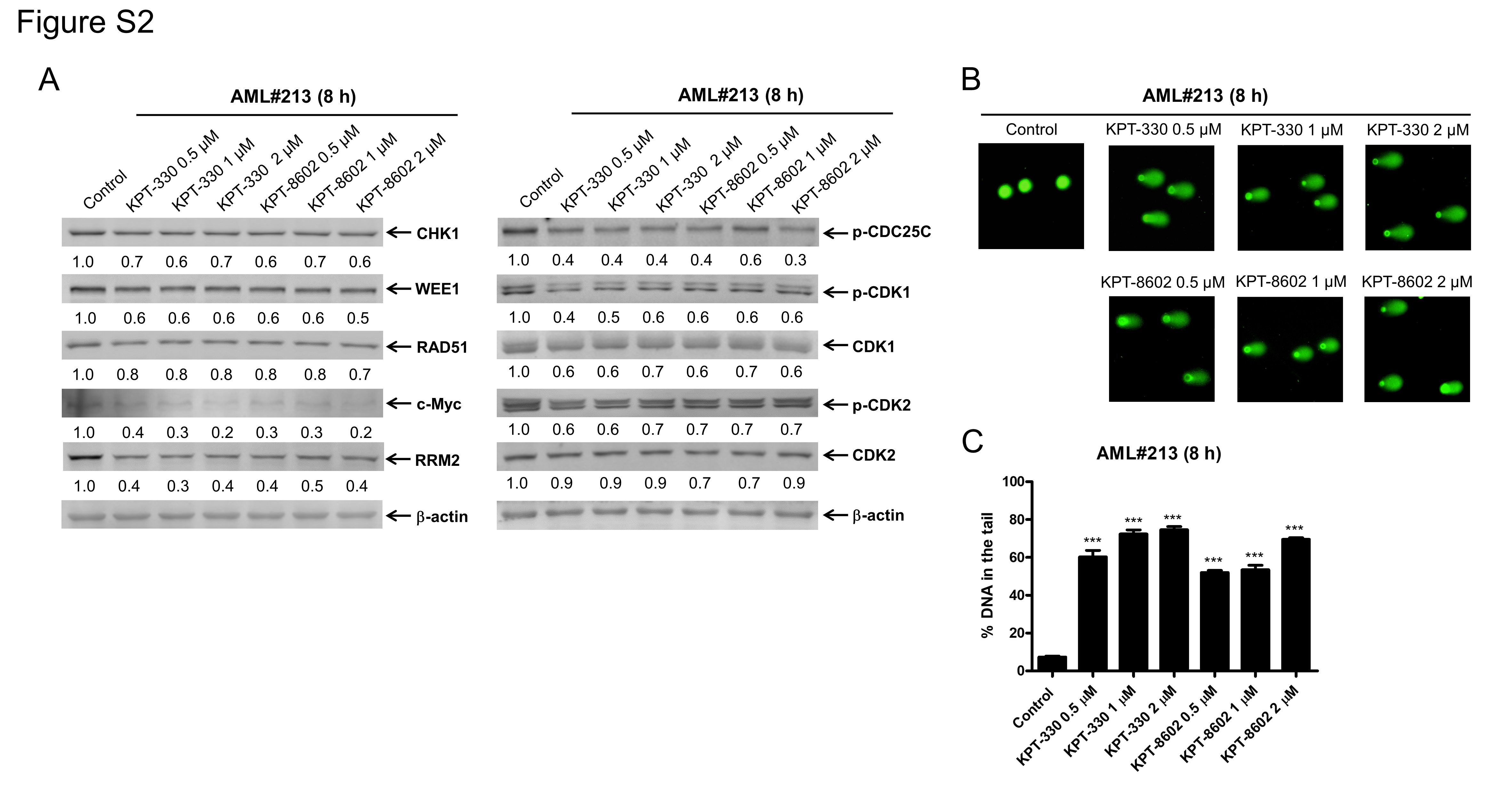

Supplement: Supplementary file 2 — Fig S2 [file JCMM-26-2646-s005.tif]

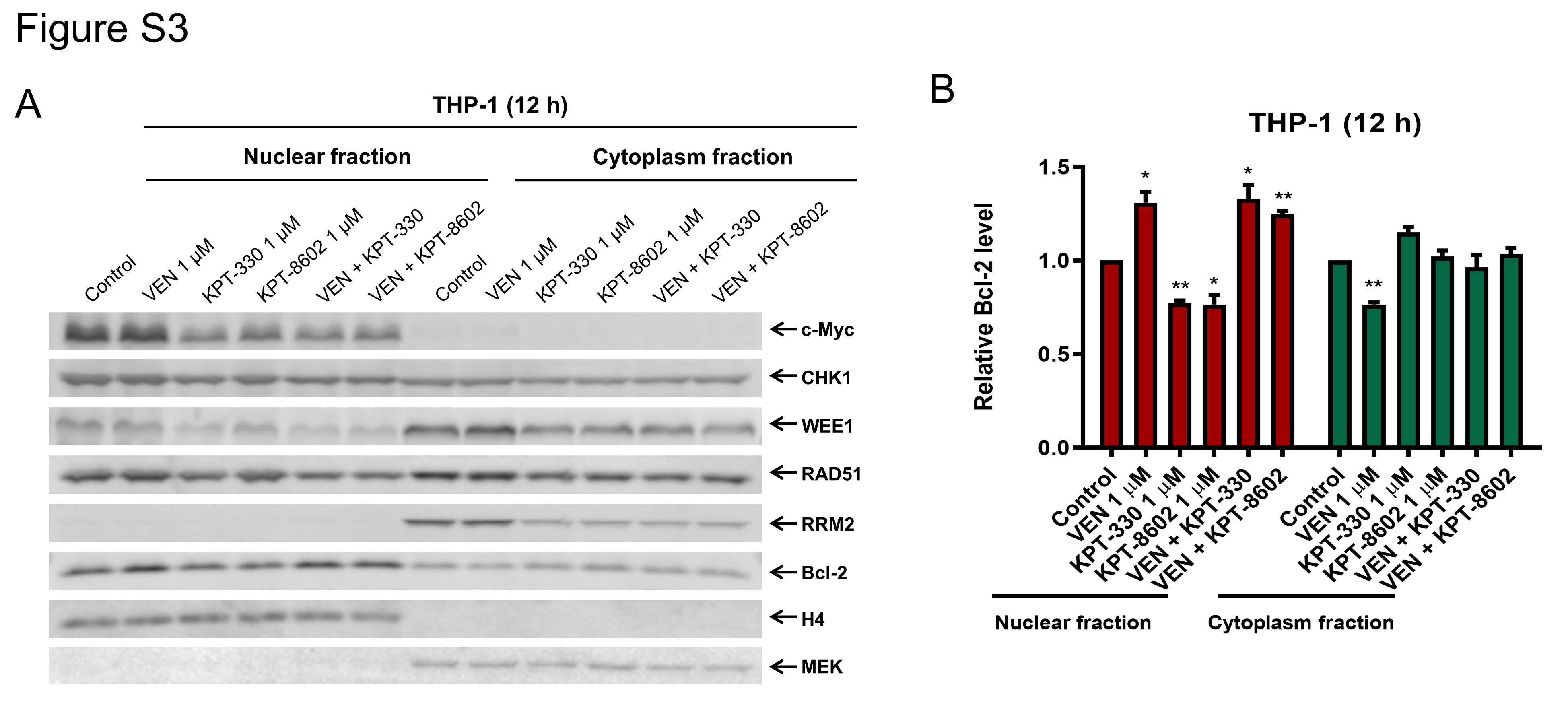

Supplement: Supplementary file 3 — Fig S3 [file JCMM-26-2646-s002.tif]
