# Supplementary material for: Venetoclax enhances DNA damage induced by XPO1 inhibitors: A novel mechanism underlying the synergistic antileukaemic effect in acute myeloid leukaemia
Source: J Cell Mol Med. 2022 Mar 31;26(9):2646–57. doi: 10.1111/jcmm.17274 (PMC9077288; doi:10.1111/jcmm.17274)
Supplement: Supplementary file 4 — Table S1 [file JCMM-26-2646-s001.doc]

**Venetoclax Enhances DNA Damage Induced by XPO1 Inhibitors: A Novel Mechanism Underlying the Synergistic Antileukemic Effect in AML**

Hanxi Yu^a^, Shuangshuang Wu^a^, Shuang Liu^a^, Xinyu Li^a^, Yuqing Gai^a^, Hai Lin^b^, Yue Wang^c^, Holly Edwards^d^, Yubin Ge^d^, and Guan Wang^a, *^

^a^ National Engineering Laboratory for AIDS Vaccine, Key Laboratory for Molecular Enzymology and Engineering, the Ministry of Education, School of Life Sciences, Jilin University, Changchun, China

^b^ Department of Hematology and Oncology, the First Hospital of Jilin University, Changchun, China

^c^ Department of Pediatric Hematology and Oncology, the First Hospital of Jilin University, Changchun, China

^d^ Department of Oncology, Molecular Therapeutics Program, Barbara Ann Karmanos Cancer Institute, Wayne State University School of Medicine, Detroit, MI, USA

*Corresponding author

Correspondence:

Guan Wang, Ph.D.

School of Life Sciences, Jilin University

2699 Qianjin Street, Changchun City, Jilin Province, P.R. China

Email: [wg10@jlu.edu.cn](mailto:wg10@jlu.edu.cn)

Table S1. Patient characteristics of primary AML patient samples.

| **Patient** | **Gender** | **Age (Year)** | **Disease Status** | **Cytogenetics** | **Blast Purity (%)** | **Gene Mutation** |
| --- | --- | --- | --- | --- | --- | --- |
| **AML#213** | **Male** | **3** | **Newly diagnosed** | **46, XY** | **64** | **FLT3-ITD** |
| **AML#237** | **Male** | **56** | **Relapsed** | **46, XY** | **88** | **FLT3-ITD, CEBPA** |
