# Supplementary material for: Venetoclax enhances DNA damage induced by XPO1 inhibitors: A novel mechanism underlying the synergistic antileukaemic effect in acute myeloid leukaemia
Source: J Cell Mol Med. 2022 Mar 31;26(9):2646–57. doi: 10.1111/jcmm.17274 (PMC9077288; doi:10.1111/jcmm.17274)
Supplement: Supplementary file 5 — Supplementary Material [file JCMM-26-2646-s003.doc]

**Venetoclax Enhances DNA Damage Induced by XPO1 Inhibitors: A Novel Mechanism Underlying the Synergistic Antileukemic Effect in AML**

Hanxi Yu^a^, Shuangshuang Wu^a^, Shuang Liu^a^, Xinyu Li^a^, Yuqing Gai^a^, Hai Lin^b^, Yue Wang^c^, Holly Edwards^d^, Yubin Ge^d^, and Guan Wang^a, *^

^a^ National Engineering Laboratory for AIDS Vaccine, Key Laboratory for Molecular Enzymology and Engineering, the Ministry of Education, School of Life Sciences, Jilin University, Changchun, China

^b^ Department of Hematology and Oncology, the First Hospital of Jilin University, Changchun, China

^c^ Department of Pediatric Hematology and Oncology, the First Hospital of Jilin University, Changchun, China

^d^ Department of Oncology, Molecular Therapeutics Program, Barbara Ann Karmanos Cancer Institute, Wayne State University School of Medicine, Detroit, MI, USA

*Corresponding author

Correspondence:

Guan Wang, Ph.D.

School of Life Sciences, Jilin University

2699 Qianjin Street, Changchun City, Jilin Province, P.R. China

Email: [wg10@jlu.edu.cn](mailto:wg10@jlu.edu.cn)

**Figure S1. Inhibition of XPO1 or c-Myc induces DNA damage in THP-1 cells.** THP-1 cells were treated with KPT-330 or KPT-8602 for 4 or 8 hours, and then subjected to apoptosis assay (panel A) and alkaline comet assay (panels B&C), respectively. (A) Cell apoptosis was assessed by Annexin V-FITC/PI staining and flow cytometry analysis. Mean percent Annexin V+ cells ± SEM are shown. Cells treated with an apoptosis inducer purchased from the Beyotime Biotechnology (Shanghai, China) were used as the positive control. (B) Representative images from alkaline comet assay are shown. (C) Alkaline comet assay results are graphed as median percent DNA in the tail from three replicate gels ± SEM. (D) THP-1 cells were treated with 10058-F4 for 4 hours, and then subjected to western blotting and alkaline comet assay, respectively. Whole cell lysates were subjected to western blotting and probed with the indicated antibodies. The fold changes for the densitometry measurements, normalized to β-actin and then compared to vehicle control, are shown below the corresponding blot (panel D). Representative images from the alkaline comet assay are shown in panel E. Alkaline comet assay results are graphed as median percent DNA in the tail from three replicate gels ± SEM (panel F). * indicates *p*<0.05, ** indicates *p*<0.01, and *** indicates *p*<0.001 compared to vehicle control.

**Figure S2. Inhibition of XPO1 induces DNA damage in a primary AML patient sample.** AML patient sample cells were treated with KPT-330 or KPT-8602 for 8 hours, and then subjected to western blot analysis (panel A) and alkaline comet assay (panels B&C), respectively. (A) Whole cell lysates were separated by SDS-PAGE, transferred onto PVDF membrane, and then probed with the indicated antibodies. The fold changes for the densitometry measurements, normalized to β-actin and then compared to vehicle control, are shown below the corresponding blot. (B) Representative images from alkaline comet assay are shown. (C) Alkaline comet assay results are graphed as median percent DNA in the tail from three replicate gels ± SEM. *** indicates *p*<0.001 compared to vehicle control.

**Figure S3. Venetoclax increases Bcl-2 nuclear localization in THP-1 cells.** THP-1 cells were treated with KPT-330, KPT-8602, and venetoclax (VEN), alone or in combination for 12 hours. Nuclear and cytoplasmic fractions were extracted and subjected to western blotting. Representative western blots are shown in panel A. The fold changes for the Bcl-2 densitometry measurements, normalized to H4 or MEK and then compared to vehicle control, are graphed as mean fold change ± SEM and shown in panel B. * indicates *p*<0.05 and ** indicates *p*<0.01 compared to vehicle control.
